# Multispectral imaging and autofluorescence photobleaching combined in a multi-head neural network for skin cancer classification

**DOI:** 10.3389/fmed.2026.1763105

**Published:** 2026-02-10

**Authors:** Vilen Jumutc, Andrey Bondarenko, Mihails Kovalovs, Ilze Lihacova, Alexey Lihachev, Dmitrijs Bļizņuks

**Affiliations:** 1 Institute of Applied Computer Systems, Riga Technical University, Riga, Latvia; 2 Institute of Atomic Physics and Spectroscopy, University of Latvia, Riga, Latvia

**Keywords:** deep learning, multi-head architecture, neural networks, photobleaching, skin cancer

## Abstract

**Introduction:**

The classification of hyperspectral images for skin cancer presents a significant challenge due to high data dimensionality and subtle differences between melanoma and non-melanoma tissues. This study investigates the efficacy of multi-target, multi-head neural network architectures for improving accuracy and precision-recall performance in hyperspectral melanoma classification.

**Methods:**

We use a proprietary multispectral dataset enriched with autofluorescence photobleaching tabular data, previously developed for skin lesion classification. Our method applies a multi-head architecture in which each head uses a different loss function, designed to optimize specific parts of the classification task. The network simultaneously learns from multiple data modalities, improving its ability to detect hidden features indicative of skin cancer. Final classifications are obtained by aggregating the outputs from all heads via simple averaging.

**Results:**

Results demonstrate significant improvements in classification accuracy and robustness compared to conventional single-head models. Our multi-head, multi-loss approach achieves the best performance on both evaluated data sources, with AUC-PR scores of 0.850 ± 0.032 and 0.822 ± 0.022 for the proprietary and ISIC datasets, respectively.

**Discussion:**

These findings indicate that multi-head architectures with specialized loss functions offer a powerful means of enhancing hyperspectral image classification, particularly for skin cancer detection, and provide a promising direction for future research and clinical applications.

## 1 Introduction

Melanoma is one of the most aggressive forms of skin cancer, accounting for the majority of skin cancer-related deaths despite representing a minority of overall cases (1, 2). Early detection is critical, as the prognosis for patients diagnosed at an early stage is significantly more favorable than for those diagnosed at advanced stages (3). Traditional diagnostic methods, including dermoscopy and histopathology, have improved substantially over recent decades but still face limitations due to inter-observer variability and the inherent subjectivity of visual assessment (4, 5). Consequently, the development of automated, objective, and reproducible diagnostic tools has emerged as a major research focus in dermatology and medical imaging (6).

Hyperspectral imaging (HSI) has gained attention as a promising non-invasive modality for skin cancer detection. Unlike conventional techniques, which capture only three color channels (red, green, blue), HSI acquires information across hundreds of contiguous spectral bands (7). This enables simultaneous extraction of spatial and spectral information, allowing differentiation of tissue types based on their unique spectral signatures (8, 9). In melanoma research, HSI has demonstrated potential for capturing subtle biochemical and morphological differences between malignant and benign lesions that may not be visible to the human eye (10).

Despite its promise, hyperspectral data classification presents significant challenges. The high dimensionality of the spectral space (often termed the “curse of dimensionality”) can lead to overfitting and poor generalization if not properly managed (11). Furthermore, hyperspectral melanoma datasets are typically limited in size due to acquisition difficulties, complicating the application and training of data-intensive deep learning models (12). Additionally, spectral differences between melanoma and other pigmented lesions are often subtle, requiring highly discriminative models capable of extracting nuanced patterns (13).

Another relevant photophysical phenomenon in skin cancer classification is autofluorescence photobleaching. When tissue is exposed to sustained 405 nm narrow-band LED excitation, endogenous fluorophores, such as NADH, collagen, and elastin, undergo photobleaching, resulting in gradual reduction of the autofluorescence signal over time (14). This process can affect the accuracy of hyperspectral or fluorescence-based diagnostic tools, particularly when image acquisition spans several seconds or longer. Quantifying this effect requires measuring autofluorescence signal intensities from melanoma lesions over multi-minute periods under continuous LED illumination.

Deep learning, particularly convolutional neural networks (CNNs), has become the state-of-the-art approach in medical image analysis due to its ability to automatically learn hierarchical features from raw data (15–17). Recent advances have demonstrated the effectiveness of CNNs in dermatological applications, including lesion segmentation, dermoscopic image classification, and multimodal fusion for melanoma detection (18, 19). However, application of CNNs to hyperspectral data remains an active research area, with unique challenges related to spectral-spatial feature extraction, model interpretability, modality combination, and performance optimization under limited data regimes (20).

One promising approach for improving classification performance is the use of multi-head neural network architectures. Unlike conventional single-head models, which optimize a single loss or combination of loss functions, multi-head networks employ multiple output branches (or “heads”), each associated with distinct objectives. This design enables simultaneous learning of complementary representations, with each head focusing on a different aspect of the classification task (21). Such architectures have been successfully applied in natural language processing (22), computer vision (23), and more recently in medical imaging (24), where they have improved robustness, generalization, and task-specific accuracy.

In the context of hyperspectral melanoma detection, multi-head architectures offer several advantages. By associating each head with a specialized combination of loss functions, the network can simultaneously optimize for metrics such as accuracy, sensitivity, and precision-recall balance. This is particularly important in clinical applications, where false negatives (missed melanomas) and false positives (benign lesions misclassified as malignant) carry significant consequences for patient outcomes (25). Moreover, aggregating outputs from multiple heads can reduce variance and improve prediction stability, offering a more reliable decision-making process compared to single-head models (26).

In this study, we use a novel hyperspectral dataset enriched with autofluorescence photobleaching tabular data, specifically curated for skin cancer classification. This dataset provides a foundation for exploring data fusion with multi-target, multi-head neural network architectures. Our approach leverages multi-head design patterns, where each head is trained with a distinct combination of loss functions tailored to optimize particular classification aspects. The final prediction is obtained through an aggregation mechanism that combines outputs from multiple heads into a consensus decision. Preliminary results indicate that this strategy yields substantial improvements in classification accuracy, precision-recall performance, and robustness over conventional single-head models.

The main contributions of this work are summarized as follows:

Proprietary hyperspectral dataset is presented. It is enriched with autofluorescence photobleaching tabular data, specifically collected for melanoma classification, augmenting the limited amount of publicly available hyperspectral medical datasets.

Multi-target with multi-head neural network framework is proposed. That is aimed for hyperspectral melanoma classification, wherein each head is guided by a distinct combination of loss functions.

Demonstration of aggregated multi-head outputs, that significantly improves classification performance compared to conventional single-head approaches.

By advancing the state of the art in hyperspectral skin cancer classification, this work highlights the potential of multi-head neural networks as robust and clinically relevant tools for early cancer detection. Our findings may inspire future research directions not only in melanoma imaging but also in other domains of medical hyperspectral analysis where high-dimensional, nuanced data are prevalent.

## 2 Related work

A variety of imaging modalities and preprocessing techniques have been explored in skin lesion analysis, particularly those combining spectral, spatial, and fluorescence information. Multispectral and autofluorescence imaging represent important approaches. For example, Lihacova et al. (27) employ spectral reflectance at several wavelengths (526 nm, 663 nm, 964 nm) plus autofluorescence under 405 nm excitation. They combine these multimodal inputs into a convolutional neural network (CNN) classifier, using data augmentation and careful channel selection (randomly dropping channels during training) to mitigate overfitting on limited datasets.

Hyperspectral imaging (HSI) methods have also been utilized, often in conjunction with deep learning models. Hirano et al. (28) describe a hyperspectral imager that captures reflectance across a wide visible-to-near-infrared range (398–757 nm), with high spectral resolution (1.45 nm steps) and spatial resolution in the tens of micrometers. They apply CNNs (GoogLeNet in particular) to classify melanoma versus non-melanoma after suitable preprocessing (e.g., calibration, wavelength selection). Similarly, Chen et al. (29) demonstrate that combining spectral diffuse reflectance and autofluorescence imaging on skin flap models enables high-accuracy identification of vascular occlusions via joint imaging spectroscopy.

Photophysical effects such as autofluorescence bleaching have also been investigated. Jakovels et al. (30) describe a protocol wherein fluorescence images are periodically captured from the same tissue region under continuous excitation; the pixel-wise decay of fluorescence is then quantified to generate spatial maps of bleaching rate, revealing local variation in photobleaching. Studies by Lesins et al. (31) examine the temporal behavior of skin autofluorescence under various excitation wavelengths (e.g., 405, 473, 532 nm), including both spectral and spatial effects. These methods are relevant to autofluorescence photobleaching data, particularly regarding measurement protocols and imaging impact.

Feature extraction and classification methods span from traditional handcrafted features and ensembles to recent spatial-spectral deep learning architectures. Deep learning is increasingly preferred for HSI classification tasks: spatial-spectral models (e.g., 3D CNNs, combined 3D-2D CNNs, wavelet CNNs) enable simultaneous learning of spectral dependencies and spatial patterns. For instance, SpectralNET blends wavelet transforms with 2D CNN layers to extract multi-resolution spectral features, followed by spatial processing for classification (32). Additionally, fusion models combining Graph Convolutional Networks (GCNs) or Graph Attention Networks (GATs) with CNNs have shown advantages, allowing representation of both pixel/superpixel relations (via graph structure) and local spatial-spectral structure (via convolution) (33).

In summary, relevant methods fall into several categories: (1) imaging modalities combining reflectance, fluorescence (autofluorescence), and/or hyperspectral bands; (2) handling photobleaching or signal decay through temporal imaging; (3) spectral-spatial feature extraction via deep models; (4) channel/wavelength selection and data augmentation to reduce overfitting; (5) architecture variations including multi-branch, multi-head, ensemble, or graph-based models. Our proposed multi-head architecture, which combines hyperspectral and autofluorescence photobleaching data, is well situated relative to these prior methods and draws on established precedents in the literature.

## 3 Materials and methods

Our approach employs a multi-head neural network design wherein each head is specifically designed to learn and optimize different aspects of the skin cancer classification problem. This architecture facilitates multi-objective optimization, allowing various data characteristics to be learned concurrently through different loss functions. The network is trained end-to-end and uses multiple data modalities and feature views, enabling finer learning and improved generalization.

### 3.1 Multi-head architecture

The model backbone consists of a shared feature extractor that learns a common representation from input dermoscopic images. We experiment with two backbones: pretrained InceptionResNetV2 (34) and randomly initialized ResNet50 (35). The use of the latter backbone is primarily motivated by an investigation into the impact of smaller, unbiased encoders on the results. Two parallel heads are appended to this shared encoder. One of the heads is fed with an additional tabular autofluorescence photobleaching input. Each head processes the shared feature map and generates predictions for different classification subtasks. Specifically:

- **Head 1** processes soft targets (approximating a probability distribution across classes), backbone-extracted features, and autofluorescence photobleaching information in tabular form. This head uses a combination of binary cross-entropy (defined per single output logit) and Kullback–Leibler divergence (defined across all classes/outputs) to efficiently propagate labeling information encoded in soft targets.
- **Head 2** focuses on standard multiclass classification with hard targets (one-hot encoded) for various skin lesion types using backbone-extracted features. This head employs a standard cross-entropy loss.

**Figure 1:**
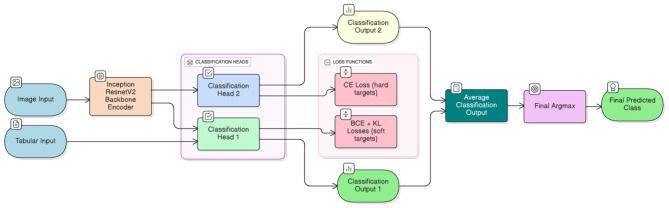
A schematic flow diagram of the multi-head architecture. From left to right, the figure depicts a typical information flow. Inputs comprise hyperspectral imaging data and tabular autofluorescence photobleaching features. Concatenated hyperspectral images are fed to the InceptionResNetV2 backbone encoder (34), and extracted features together with tabular data are processed by different classification heads. Finally, classification outputs are averaged to produce the final prediction. Different loss functions are outlined to enable clear distinction between heads and facilitate use of differently treated classification targets.

All abovementioned heads are implemented as multi-layer perceptrons (MLPs) connected either to the shared feature map (Head 2) or to a concatenation of shared features and tabular inputs (Head 1). The overall neural network architecture with pretrained InceptionResNetV2 backbone is illustrated in Figure 1.

### 3.2 Loss functions

Each classification head in Figure 1 is optimized using a single or combination of loss functions tailored to its specific task. Diverse loss functions enable the model to capture richer signals from the data.

For the first multiclass classification head, we use standard categorical cross-entropy loss, defined as:


$$\begin{array}{l} L_{\text{CE}}=-\overset{C}{\underset{i=1}{\sum }}y_{i}\log\left({ŷ}_{i}\right), \end{array}$$ (1)

where *C* is the number of classes, *y*_*i*_ is the ground-truth label (one-hot encoded), and ŷ_*i*_ is the predicted probability for class *i*. This loss penalizes incorrect predictions and encourages high probability for the correct class.

For the binary classification task with soft targets, we use binary cross-entropy, suitable for two-class problems. It is defined as:


$$\begin{array}{l} L_{\text{BCE}}=-\left[y\log\left(ŷ\right)+\left(1-y\right)\log\left(1-ŷ\right)\right], \end{array}$$ (2)

where *y*∈[0, 1] is the ground-truth soft label and ŷ∈[0, 1] is the predicted probability of the target class. This loss is effective for binary decision-making scenarios with imbalanced datasets.

To encourage distributional alignment and improve output calibration, one head is additionally trained using Kullback–Leibler (KL) divergence, which measures divergence between the predicted distribution $\hat{P}$ and a target distribution *Q* (represented by soft labels, see details in Section 4.1):


$$\begin{array}{l} L_{\text{KL}}=\overset{C}{\underset{i=1}{\sum }}Q\left(i\right)\log\left(\frac{Q\left(i\right)}{\hat{P}\left(i\right)}\right) \end{array}$$ (3)

This loss is particularly useful when the model must match certain label priors or leverage knowledge distillation techniques.

### 3.3 Loss aggregation and training objective

The total training loss $L_{\text{total}}$ is defined as a weighted sum of individual head losses:


$$\begin{array}{l} L_{\text{total}}=\alpha L_{\text{CE}}+\beta L_{\text{BCE}}+\gamma L_{\text{KL}}, \end{array}$$ (4)

where α, β, and γ are hyperparameters controlling each head's contribution to the total objective. Ideally, these weights should be tuned based on validation performance. Considering the scope of our study and extensive experimentation, we fixed all loss weights to α = β = γ = 1.

### 3.4 Prediction aggregation

During inference, outputs from all heads are aggregated to produce the final classification decision. We utilize simple averaging over outputs:


$$\begin{array}{l} {ŷ}_{\text{final}}=\frac{1}{N}\overset{N}{\underset{i=1}{\sum }}{ŷ}^{\left(i\right)}, \end{array}$$ (5)

where *N* is the number of heads and ŷ^(*i*)^ is the prediction from the *i*-th head. This ensembling mechanism enhances robustness and leverages diversity among individual heads.

### 3.5 Datasets

#### 3.5.1 Multispectral proprietary dataset

The primary focus of this study is a multispectral dataset obtained from (27). Dataset is constantly updated with new lesions and currently has more than 4,000 lesions. Since not all past images have autflourescence images sequence, we kept 2,066 skin lesions to investigate photobleaching dynamics under controlled multispectral illumination. Each lesion was taken using a portable multispectral device equipped with narrow-band LEDs centered at 405 nm (autofluorescence excitation), 526 nm (green reflectance), 663 nm (red reflectance), and 964 nm (near-infrared reflectance). Long pass filter placed to block the reflected excitation 405 nm light from the skin surface. The final dataset contains 36 distinct classes of lesions grouped into 3 larger categories for the final model training. Melanoma category accounts for C43 and D03.9 classes. Malignant category comprises C44, C46 and D09 classes as well as Keratoacanthoma lesions. All the rest classes are categorized as Other. The overall distribution of major category classes can be seen in Table 1.

**Table 1:** Proprietary dataset distribution of classes.

**Category**	**No. of examples**
Melanoma	562
Malignant	377
Other	1,127

**Figure 2:**
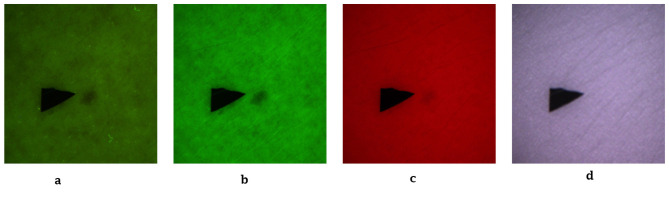
Representative non-melanoma skin images. **(a)** Autofluorescence emission image. **(b)** Green reflectance image. **(c)** Red reflectance image. **(d)** Near-IR reflectance image.

**Figure 3:**
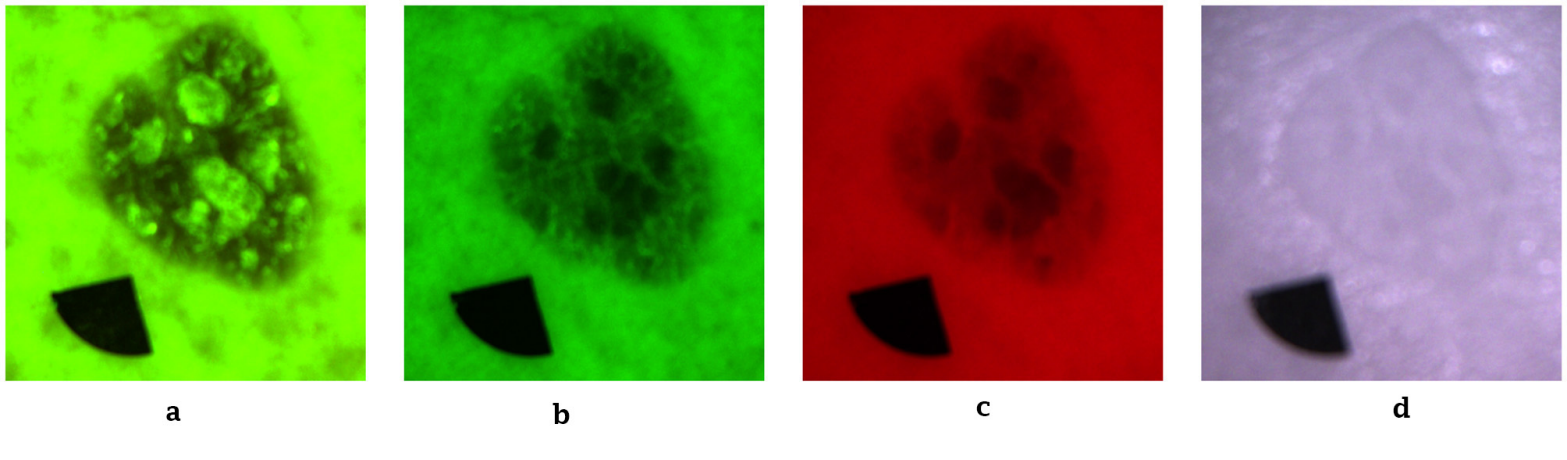
Representative melanoma skin images. **(a)** Autofluorescence emission image. **(b)** Green reflectance image. **(c)** Red reflectance image. **(d)** Near-IR reflectance image.

To minimize optical artifacts, the imaging system utilized a cross-polarization configuration, with the illumination source polarization orthogonal to that of the camera lens polarizer. This setup effectively suppressed specular reflection from the skin surface, enabling reliable measurement of diffusely reflected and fluorescent signals. All images were captured using an IDS uEye color camera. Representative non-melanoma images are shown in Figure 2, with melanoma images in Figure 3.

#### 3.5.2 Photobleaching tabular features

The second input to our multi-head architecture derives from autofluorescence photobleaching analysis. This section describes tabular data acquisition and preprocessing based on UMAP embeddings (36, 37), generated from measurements of skin images under autofluorescence excitation.

For each lesion, a sequence of 10 autofluorescence images was recorded at 1-s intervals with 500 ms exposure time, enabling temporal observation of fluorescence decay. Each frame was processed independently using a Mask R-CNN segmentation model (38) trained to delineate both lesion regions and reference markers. Lesion and marker masks were merged and inverted to derive skin background masks representing surrounding non-lesion tissue.

**Figure 4:**
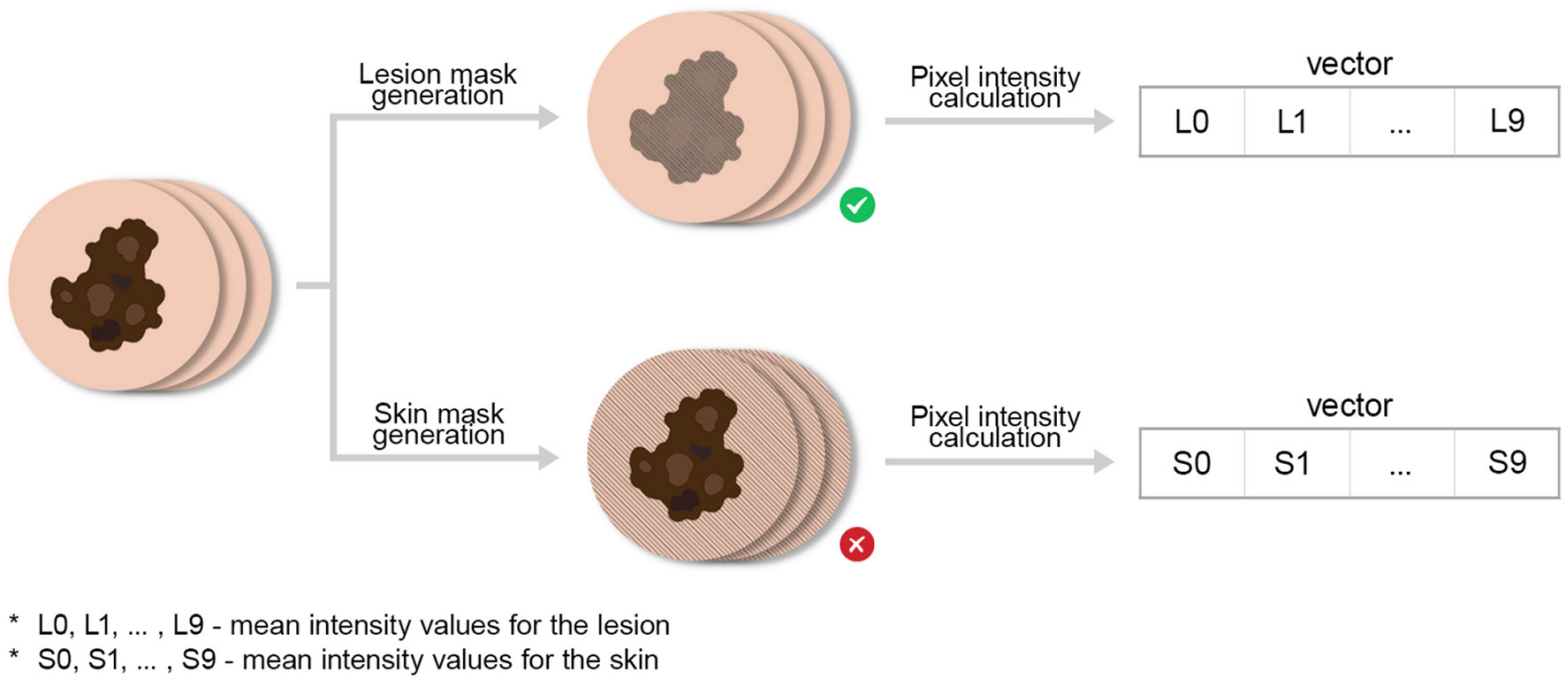
Data acquisition process for obtaining photobleaching features. We emphasize by ⊗ the abolishment of skin intensity features (for the analysis) which were co-generated during this process.

**Figure 5:**
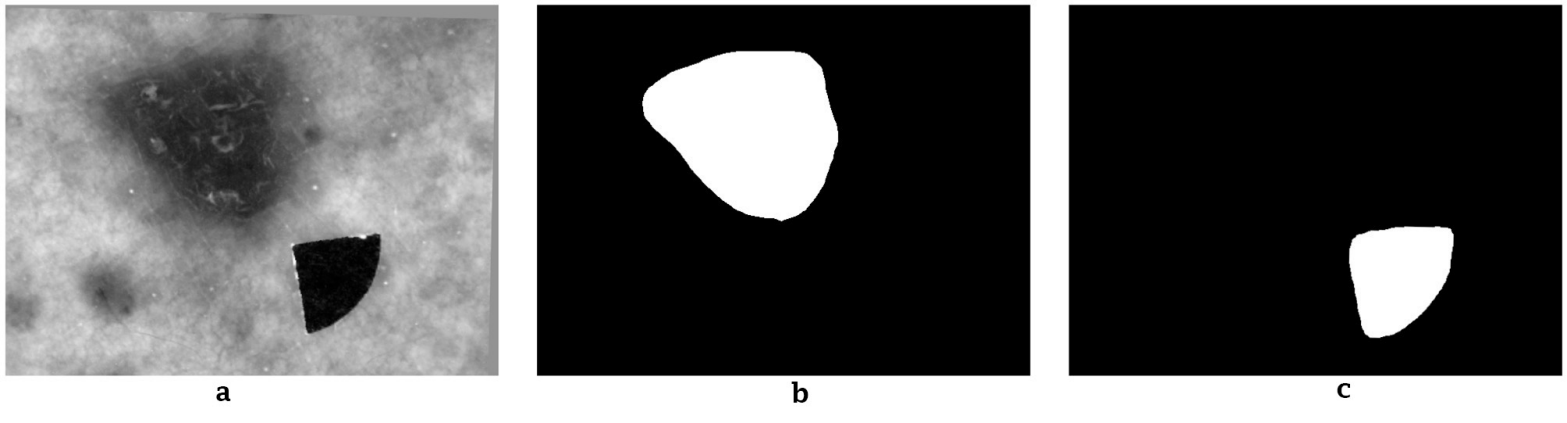
Representative lesion image with segmentation masks for lesion and marker. **(a)** Initial image. **(b)** Lesion mask. **(c)** Marker mask.

Mean pixel intensities were computed within each frame for both lesion and skin regions, yielding time-dependent fluorescence intensity profiles. Mean values from the 10 sequential images formed a 10-element feature vector representing each lesion's photobleaching curve. These vectors served as input features for model training. Only lesion intensity values were retained, as surrounding skin regions did not exhibit diagnostically valuable photobleaching behavior. The overall data acquisition process is depicted in Figure 4. A typical image example with segmentation masks is shown in Figure 5.

#### 3.5.3 UMAP-based clustering and evaluation of photobleaching features

To assess whether photobleaching-derived features can distinguish between skin lesion types and to guide the selection of informative inputs for the classification model, we applied Uniform Manifold Approximation and Projection (UMAP) (36, 37) as an nonlinear dimensionality reduction and analysis tool. Training the full multi-head classification model is computationally expensive, particularly when evaluating multiple configurations of temporal photobleaching inputs. In contrast, UMAP enables rapid exploration of feature separability and clustering behavior, making it well suited for efficiently selecting the number and composition of photobleaching images to be used in downstream classification. Accordingly, UMAP was employed to evaluate not only how many initial frames from the photobleaching sequence should be retained, but also different sequential combinations of frames drawn from other parts of the feature vector. The objective was to identify subsets of photobleaching measurements that maximize class separability while minimizing redundant or noise-dominated information before subsequent classifier training.

UMAP models were trained using supervised fitting, with each measurement assigned a class label corresponding to its histopathological diagnosis (only a subset of initial classes was used for analysis). Some of lesions where removed since they had less than 10 flourescence images (proposed AI model can handle such situations).

**Figure 6:**
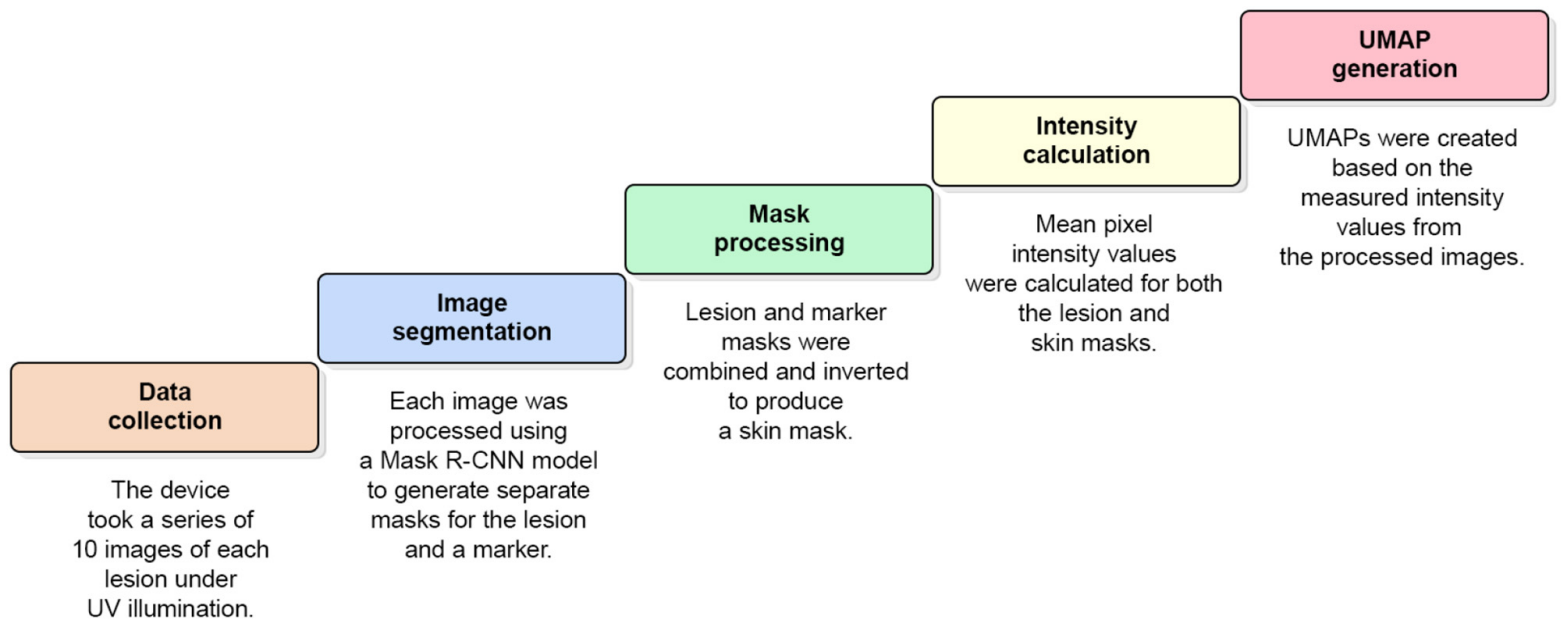
Data acquisition process for generating UMAP embeddings.

The overall embedding generation process is shown in Figure 6. Our goal was to obtain clearly defined, separable clusters for each lesion class. We systematically tested a wide range of UMAP parameters (including n_neighbors, min_dist, metric, and n_components) and distance metrics. For each parameter set, we evaluated embeddings using a purity clustering quality metric, which is defined as the average fraction of each point's *k*-nearest neighbors belonging to the same class. This reflects local class consistency within the embedding, similar to previously proposed metrics for evaluating dimensionality reduction (36, 37, 39, 40). The corresponding formula can be found below:


$$\begin{array}{c} Purity\left(k\right)=\frac{1}{N}\overset{N}{\underset{i=1}{\sum }}\frac{1}{k}\underset{j\in N_{k}\left(i\right)}{\sum }\text{1}\left\{y_{j}=y_{i}\right\}, \end{array}$$ (6)

where:

- *N* is the total number of points
- $N_{k}\left(i\right)$ is the set of the *k*-nearest neighbors of point *i*
- *y*_*i*_ is the class label of point *i*
- **1**{·} is the indicator function.

Initially, both lesion and skin mean intensity features were evaluated. However, comparisons across parameter sets revealed that skin bleaching did not improve clustering quality and, in some cases, introduced noise. Consequently, only lesion-derived features were retained.

To determine how the number of consecutive images from each photobleaching sequence affects class separability, we trained UMAP models using increasing subsets of the 10 available images per lesion. Clustering quality metrics revealed that optimal purity scores were achieved using only the first four images. Including additional frames beyond this point caused gradual performance decline. We attribute this to saturation of the photobleaching process the most pronounced autofluorescence intensity decrease occurs during initial illumination seconds, after which the signal stabilizes and becomes noise-dominated. Later images thus carry little diagnostic information and may obscure meaningful inter-class variance.

**Figure 7:**
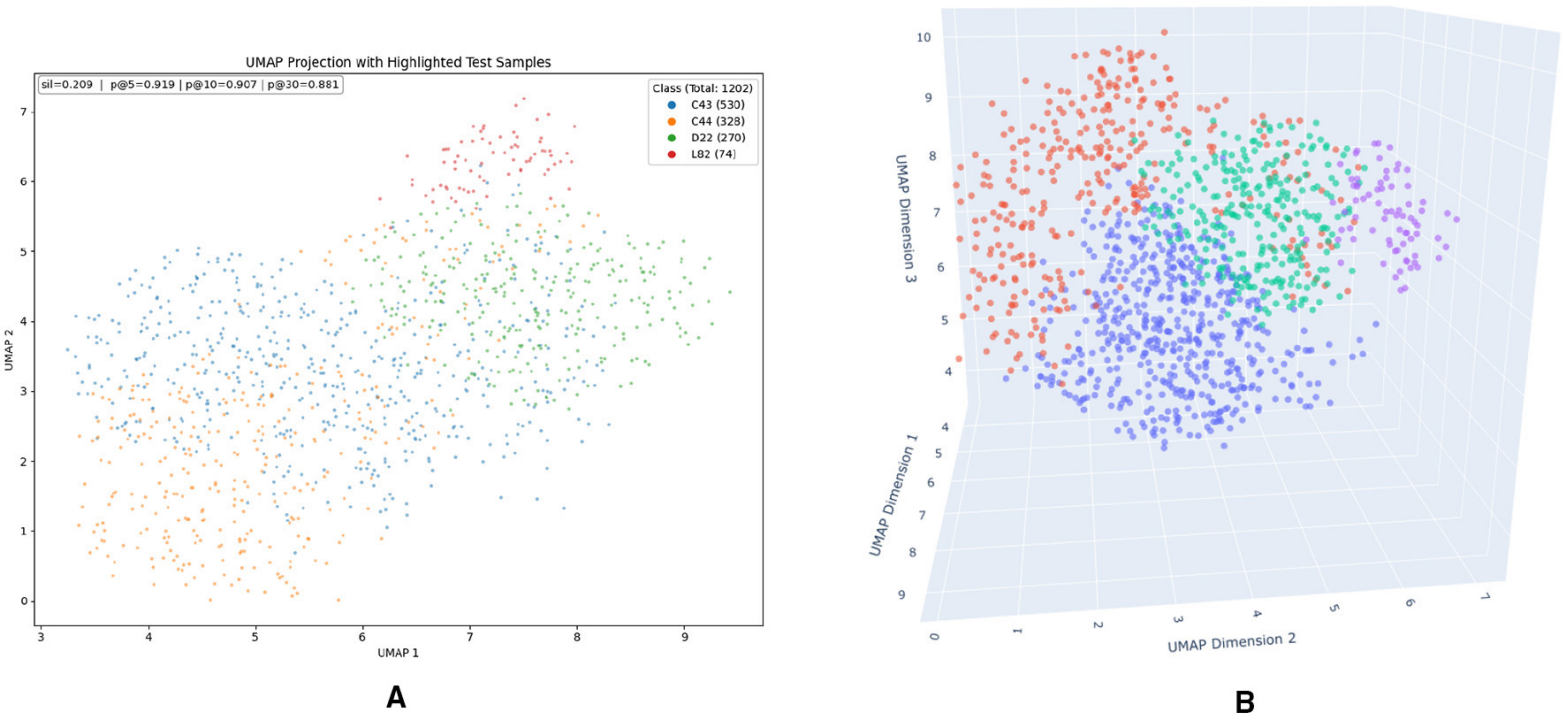
UMAP projections in 2D and 3D dimensions. **(a)** 2D projection (matplotlib). **(b)** 3D interactive projection (plotly).

The optimal UMAP configuration used the “hamming” distance for calculating the purity metric in Equation 6, yielding the best purity score of 0.978 with *k* = 5 at parameter settings n_neighbors = 150, min_dist = 0.01, and n_components = 3. Resulting embeddings are shown in Figure 7. While the 2D projection provides limited visual separation, the interactive 3D UMAP projection reveals distinct clusters corresponding to each lesion class, confirming that extracted photobleaching features capture class-relevant patterns. These findings indicate that temporal fluorescence decay during early bleaching carries meaningful diagnostic information and can serve as valuable input for downstream AI-based classification models.

#### 3.5.4 ISIC public dataset

The International Skin Imaging Collaboration (ISIC) public dataset (41) is one of the largest and most widely used collections of dermoscopic (RGB) images for skin lesion study and diagnosis, including melanomas. Created to support dermatology research and computer-aided skin cancer detection, it provides a standardized, curated set of high-quality clinical images. The dataset contains tens of thousands of images from multiple international clinical centers, ensuring diversity in skin tones, lesion types, and imaging conditions. Each image is accompanied by detailed metadata such as lesion diagnosis, patient demographics, and, in some cases, segmentation masks or lesion boundaries.

ISIC serves as the foundation for major challenges and benchmarks in automated melanoma detection, such as the annual ISIC Challenges hosted on platforms like Kaggle and at the MICCAI conference. These challenges encourage algorithm development for lesion classification, segmentation, and diagnosis. Our ISIC usage comprises a subsample of 50,000 images spanning three major classes analogous to our proprietary dataset: “benign”, “intermediate,” and “malignant”.

#### 3.5.5 Mapping between datasets

The following label mapping is applied between the datasets: ISIC “malignant” is mapped to the proprietary “Melanoma” class, ISIC “intermediate” to the proprietary “Malignant” (non-melanoma) class, and ISIC “benign” to the proprietary “Other” class. Although the proprietary “Malignant” category includes ICD-coded malignant and *in situ* diagnoses (C44, C46, D09), such lesions are commonly treated as a distinct risk group in ISIC-based classification schemes. In these schemes, the ISIC “malignant” class predominantly represents melanoma, while non-melanoma malignancies and premalignant lesions are grouped under an “intermediate” risk category. Accordingly, we adopt a risk-level mapping between datasets rather than a strict histopathological correspondence. Finally, we note that photobleaching features were not used for the ISIC dataset because they are unavailable, and that the tabular input for Classification Head 1 was not concatenated with the shared visual feature map (see Figure 1).

## 4 Results

### 4.1 Experimental setup

In all experiments with the proprietary melanoma dataset, we employed five fixed random cross-validation splits into training, validation, and test folds of 80%, 10%, and 10% of total samples, respectively. For the ISIC dataset, a single split was generated. Training was repeated three times per split, yielding 15 total training runs for the proprietary dataset and three for ISIC. All images were self-normalized to pixel values in [0, 1] interval and down- or upscaled to 448 × 448 spacial dimensions. We have applied very basic training augmentation techniques, i.e., adding gamma, contrast and gaussian noise to the input images and only gaussian noise to the tabular photobleaching inputs. Soft labels for BCE and KL losses were precomputed depending on the target class, i.e., for Melanoma class it was: [0.7, 0.2, 0.1]; for Malignant class: [0.2, 0.7, 0.1]; and finally for Other class: [0.1, 0.1, 0.8]. The difference in probability mass allocation is attributed to inherent similarities between Melanoma and Malignant, while both of them being equally distant from Other class.

Training ran for a maximum of 50 epochs for the proprietary dataset and 10 epochs for ISIC. We monitored validation loss decay each epoch and employed a “reduce-on-plateau” learning rate scheduler with factor 0.5 and patience of 5 epochs for the proprietary dataset (2 epochs for ISIC). For final test set evaluation, we used the best checkpointed model (selected based on validation loss).

All experiments used the Nadam (42) optimizer with initial learning rate 0.0002 and weight decay 0.0001. Batch size was set to 4 for all datasets. Training was performed on an Nvidia RTX 4090 GPU with 24GB RAM. Code was developed in Python 3.10.15 using the fuse-med-ml framework (43). The experimental code is available at fuse-melanoma repository.

The InceptionResNetV2 backbone was initialized from a pretrained checkpoint (43) except for the first layer, which has 12 channels for our proprietary dataset. The ResNet50 backbone was randomly initialized. All MLP layers in classification heads were also randomly initialized.

### 4.2 Main results

In our experiments, we track two primary metrics for comparability: overall Accuracy and weighted (across classes) AUC-PR (Area Under the Precision-Recall Curve). The multi-class classification accuracy is defined as


$$\text{Accuracy}=\frac{1}{N}\overset{N}{\underset{i=1}{\sum }}\text{1}\left({ŷ}_{i}=y_{i}\right),$$

where *N* denotes the total number of samples, *y*_*i*_ is the true class label of the *i*-th sample, ŷ_*i*_ is the predicted class label, and **1**(·) is the indicator function, which equals 1 if its argument is true and 0 otherwise.

AUC-PR is defined as the area under the Precision-Recall curve:


$$\begin{array}{c} {\text{AUC}}_{PR}=\int_{0}^{1}P\left(R\right)dR \end{array}$$ (7)

where Precision (*P*) and Recall (*R*) are given by:


$$\begin{array}{c} P=\frac{TP}{TP+FP},\;R=\frac{TP}{TP+FN}, \end{array}$$ (8)

and *TP* = True Positives, *TN* = True Negatives, *FP* = False Positives and finally *FN* = False Negatives. In the discrete (empirical) case, AUC-PR can be approximated using the trapezoidal rule:


$$\begin{array}{c} {\text{AUC}}_{PR}=\overset{n-1}{\underset{i=1}{\sum }}\left(R_{i+1}-R_{i}\right)\cdot \frac{P_{i+1}+P_{i}}{2}. \end{array}$$ (9)

Tables 2, 3 report main test set results across datasets, comparing our architecture to single-head classification with pretrained or randomly initialized backbones. All scores include standard deviations for statistical comparison. For completeness, we also compare different loss function combinations for single-head architectures.

**Table 2:** Test set performance for the proprietary dataset.

**No**.	**Dataset note**	**Model**	**Losses**	**Accuracy**	**AUC-PR**
1	No tabular inputs	Multi-Head InceptionResNetV2 (ours)	CE, BCE & KL	0.798 ± 0.036	0.850 ± 0.032
2	No tabular inputs	Multi-Head InceptionResNetV2 (ours)	CE, BCE	0.789 ± 0.035	0.838 ± 0.032
3	No tabular inputs	Single-Head InceptionResNetV2	CE, BCE & KL	0.768 ± 0.030	0.823 ± 0.039
4	No tabular inputs	Single-Head InceptionResNetV2	CE, BCE	0.786 ± 0.036	0.830 ± 0.036
5	With 3 tabular inputs	Multi-Head InceptionResNetV2 (ours)	CE, BCE & KL	0.781 ± 0.038	0.836 ± 0.031
6	With 3 tabular inputs	Multi-Head InceptionResNetV2 (ours)	CE, BCE	0.772 ± 0.035	0.828 ± 0.022
7	With 3 tabular inputs	Single-Head InceptionResNetV2	CE, BCE & KL	0.780 ± 0.029	0.809 ± 0.035
8	With 3 tabular inputs	Single-Head InceptionResNetV2	CE, BCE	0.759 ± 0.041	0.784 ± 0.047
9	No tabular inputs	Multi-Head ResNet50 (ours)	CE, BCE & KL	0.770 ± 0.047	0.826 ± 0.037
10	No tabular inputs	Multi-Head ResNet50 (ours)	CE, BCE	0.765 ± 0.045	0.814 ± 0.056
11	No tabular inputs	Single-Head ResNet50	CE, BCE & KL	0.758 ± 0.046	0.825 ± 0.027
12	No tabular inputs	Single-Head ResNet50	CE, BCE	0.755 ± 0.054	0.815 ± 0.037
13	With 3 tabular inputs	Multi-Head ResNet50 (ours)	CE, BCE & KL	0.774 ± 0.052	0.817 ± 0.048
14	With 3 tabular inputs	Multi-Head ResNet50 (ours)	CE, BCE	0.752 ± 0.059	0.809 ± 0.045
15	With 3 tabular inputs	Single-Head ResNet50	CE, BCE & KL	0.746 ± 0.050	0.791 ± 0.045
16	With 3 tabular inputs	Single-Head ResNet50	CE, BCE	0.751 ± 0.041	0.786 ± 0.045

Bold values indicate the best achieved results per metric and model types.

**Table 3:** Test set performance for the ISIC dataset.

**Model**	**Losses**	**Accuracy**	**AUC-PR**
Multi-Head InceptionResNetV2 (ours)	CE, BCE & KL	0.981 ± 0.000	0.822 ± 0.022
Multi-Head InceptionResNetV2 (ours)	CE, BCE	0.982 ± 0.002	0.820 ± 0.011
Single-Head InceptionResNetV2	CE, BCE & KL	0.982 ± 0.001	0.807 ± 0.004
Single-Head InceptionResNetV2	CE, BCE	0.981 ± 0.001	0.801 ± 0.032
Multi-Head ResNet50 (ours)	CE, BCE & KL	0.976 ± 0.002	0.679 ± 0.016
Multi-Head ResNet50 (ours)	CE, BCE	0.975 ± 0.001	0.684 ± 0.017
Single-Head ResNet50	CE, BCE & KL	0.977 ± 0.001	0.677 ± 0.008
Single-Head ResNet50	CE, BCE	0.976 ± 0.000	0.675 ± 0.002

Bold values indicate the best achieved results per metric and model types.

Results demonstrate that our multi-head approach using multiple losses consistently outperforms alternatives across backbones and datasets in both metrics. The pre-trained InceptionResNetV2 backbone improves results for all inspected models on average. Conversely, the addition of tabular data improves accuracy only for the ResNet50 backbone. The evaluation of the optimal number of tabular inputs, as guided by the UMAP analysis, is performed in the ablation study and presented in Section 4.3.

### 4.3 Ablation study

This section investigates the importance of multiple loss functions for different classification heads and examines the optimal number of tabular inputs for melanoma classification.

Table 4 shows that our chosen loss combination outperforms alternatives, attributable to mutual contributions from all three losses working on differently shaped targets (soft versus hard labels).

**Table 4:** Test set performance for different loss function combinations on a single-head model (ISIC dataset).

**Backbone**	**Losses**	**Accuracy**	**AUC-PR**
InceptionResNetV2	CE, BCE & KL	0.982 ± 0.001	0.807 ± 0.004
InceptionResNetV2	CE, BCE	0.981 ± 0.001	0.801 ± 0.032
InceptionResNetV2	CE, KL	0.980 ± 0.001	0.780 ± 0.020
InceptionResNetV2	CE	0.980 ± 0.001	0.792 ± 0.014

Bold values indicate the best achieved results per metric and model types.

Table 5 reveals that performance may increase with additional tabular inputs for some models, while decreasing for others (e.g., InceptionResNetV2). Notably, accuracy and AUC-PR respond differently to the number of inputs, sometimes changing in opposite directions. When interpreted in the context of the UMAP-based analysis used to guide the selection of photobleaching inputs, these results suggest that for pretrained models, additional UMAP-selected features may introduce more noise than signal, whereas for randomly initialized smaller models, UMAP-guided input selection and additional tabular inputs remain beneficial. This behavior can be further understood in the context of negative transfer in learning with pre-trained models (44, 45).

**Table 5:** Test set performance for different numbers of tabular inputs (proprietary dataset).

**Model**	**No. of inputs**	**Losses**	**Accuracy**	**AUC-PR**
Multi-Head InceptionResNetV2	3	CE, BCE & KL	0.781 ± 0.038	0.836 ± 0.031
Multi-Head InceptionResNetV2	3	CE, BCE	0.772 ± 0.035	0.828 ± 0.022
Multi-Head InceptionResNetV2	5	CE, BCE & KL	0.776 ± 0.051	0.823 ± 0.044
Multi-Head InceptionResNetV2	5	CE, BCE	0.788 ± 0.038	0.826 ± 0.030
Multi-Head InceptionResNetV2	10	CE, BCE & KL	0.774 ± 0.037	0.819 ± 0.041
Multi-Head InceptionResNetV2	10	CE, BCE	0.784 ± 0.035	0.831 ± 0.038
Multi-Head ResNet50	3	CE, BCE & KL	0.774 ± 0.052	0.817 ± 0.048
Multi-Head ResNet50	3	CE, BCE	0.752 ± 0.059	0.809 ± 0.045
Multi-Head ResNet50	5	CE, BCE & KL	0.768 ± 0.044	0.820 ± 0.033
Multi-Head ResNet50	5	CE, BCE	0.755 ± 0.052	0.815 ± 0.043
Multi-Head ResNet50	10	CE, BCE & KL	0.771 ± 0.049	0.825 ± 0.039
Multi-Head ResNet50	10	CE, BCE	0.771 ± 0.055	0.819 ± 0.041

Bold values indicate the best achieved results per metric and model types.

### 4.4 Significance tests

To validate our experimental findings and assess their statistical significance, we conducted a series of *t*-tests for pairwise comparisons between runs, using main metric values across all folds as inputs. We report the *t*-statistic and corresponding *p-*values from independent samples *t*-tests (46), along with Cohen's d (effect size) (47) to quantify the magnitude of difference between two group means in terms of standard deviations. Table 6 presents these values for our proprietary dataset, comparing runs with our best-performing Multi-Head architectures against equivalent Single-Head configurations.

**Table 6:** Significance test results for the proprietary dataset.

**Comparison note**	**Backbone**	**Metric**	**T-statistic**	***P*-value**	**Cohen's d**
Multi-Head (1) vs. Single-Head (3)	InceptionResNetV2	Accuracy	2.438122	0.021365	0.890276
Multi-Head (1) vs. Single-Head (3)	InceptionResNetV2	AUC-PR	1.924775	0.064473	0.702829
Multi-Head (9) vs. Single-Head (11)	ResNet50	Accuracy	0.644426	0.524544	0.235311
Multi-Head (9) vs. Single-Head (11)	ResNet50	AUC-PR	0.035264	0.972119	0.012877
Multi-Head (13) vs. Single-Head (15)	ResNet50	Accuracy	1.457555	0.156088	0.532224
Multi-Head (13) vs. Single-Head (15)	ResNet50	AUC-PR	1.491648	0.146973	0.544673

As shown in Table 6, many of our best average scores yield low *p*-values, indicating statistically significant performance improvements over the Single-Head architecture. However, the ResNet50 *p*-values are considerably less significant than those for the InceptionResNetV2 backbone, suggesting limitations of this CNN architecture in producing substantially differentiated results.

## 5 Discussion

### 5.1 Challenges and future directions

Despite promising results achieved with our multi-head architecture, several challenges warrant careful consideration and present opportunities for future research.

**Dataset Limitations and Generalization:** Our proprietary dataset, while novel and carefully curated, contains more than 2,000 lesions that have autofluorescence image sequence. Still being relatively modest by deep learning standards. The limited sample size poses overfitting risks, particularly given hyperspectral data dimensionality. Furthermore, the dataset may not fully represent the diversity of skin tones, lesion locations, and disease subtypes encountered in global clinical practice. Future work should expand the dataset through multi-center collaborations and implement federated learning approaches that leverage data from multiple institutions without compromising patient privacy.

**Photobleaching Variability:** The autofluorescence photobleaching phenomenon exhibits significant inter- and intra-patient variability depending on factors such as skin pigmentation, lesion thickness, and individual fluorophore composition. Our current approach uses a fixed acquisition protocol, yet optimal imaging parameters may vary across patients. Developing adaptive acquisition strategies and normalization techniques to account for these variations could improve robustness.

**Model Interpretability:** While multi-head architectures improve performance, they also increase model complexity, complicating clinical interpretation. For dermatologists to trust and adopt these systems, better visualization tools are needed to explain which spectral bands and spatial regions contribute most to classification decisions. Integrating attention mechanisms and class activation mapping techniques specifically designed for hyperspectral data would enhance interpretability.

**Computational Efficiency:** Real-time clinical deployment requires efficient inference. Our current implementation, while feasible for research, may be too slow for high-throughput screening. Model compression techniques, pruning, and knowledge distillation approaches could maintain performance while reducing computational overhead.

**Multi-Modal Integration:** Our work combines hyperspectral imaging with photobleaching tabular data, but additional modalities could be incorporated, such as dermoscopic RGB images, clinical metadata (patient age, lesion location), and even genomic data. Developing sophisticated fusion strategies that handle heterogeneous data types without one modality dominating the learning process represents an important future direction.

**Clinical Validation and Regulatory Pathways:** Translating this research into clinical practice requires rigorous validation studies comparing system performance against expert dermatologists in real-world settings. Navigating regulatory approval for AI-based medical devices will require extensive documentation of model robustness, failure mode analysis, and bias assessment across demographic groups.

**Standardization:** The field would benefit from standardized protocols for hyperspectral image acquisition, calibration, and photobleaching measurement. Establishing community benchmarks and open-source preprocessing pipelines would accelerate progress and enable fair method comparison.

**Advanced Architectures:** While our multi-head design shows promise, exploring sophisticated ensemble strategies beyond simple averaging, such as learned gating mechanisms or attention-based aggregation could further improve performance. Graph neural networks that explicitly model spectral correlations or spatial relationships between superpixels represent another promising avenue.

**Uncertainty Quantification:** Providing calibrated uncertainty estimates alongside predictions is crucial for clinical decision-making. Bayesian deep learning approaches or ensemble methods could be adapted to our multi-head framework to quantify model confidence, helping clinicians identify cases requiring expert review.

Addressing these challenges will be essential for realizing the full potential of hyperspectral imaging and multi-head neural networks in routine melanoma detection and other dermatological applications.

### 5.2 Conclusions

This study demonstrates that multi-head neural network architectures, combined with specialized loss functions and multimodal data fusion, enhance hyperspectral melanoma classification performance compared to conventional single-head approaches (under certain model architectures and metrics). Key contributions include a novel proprietary dataset integrating multispectral imaging with autofluorescence photobleaching tabular data, and systematic evaluation of multi-head designs optimizing complementary classification objectives.

Experimental results on both our proprietary dataset and the ISIC dataset demonstrate consistent improvements in accuracy and AUC-PR across different backbone architectures. Ablation studies indicate that leveraging multiple loss functions that target both hard and soft labels yields superior performance, while the effectiveness of photobleaching feature integration is dependent on model capacity. Notably, the pretrained InceptionResNetV2 backbone consistently outperformed the randomly initialized ResNet50, highlighting the continued value of transfer learning even when adapting models to hyperspectral inputs. Our use of a randomly initialized ResNet50 was specifically motivated by an investigation into the impact of smaller, unbiased encoders on overall performance.

These findings validate our hypothesis that multi-head architectures can effectively learn richer, more discriminative representations from high-dimensional medical imaging data. By aggregating predictions from multiple specialized heads, our approach reduces prediction variance and improves robustness, critical properties for clinical applications where diagnostic reliability is paramount.

While this work focuses on melanoma detection, the proposed framework is readily adaptable to other medical imaging tasks involving complex, multimodal data. Future efforts will concentrate on clinical validation, model interpretability, and expanding dataset diversity to support broader deployment. Ultimately, this research represents a meaningful step toward objective, reproducible, and accurate AI-assisted skin cancer diagnosis, with potential to improve early detection rates and patient outcomes in dermatological care.

## Data Availability

The raw data supporting the conclusions of this article will be made available by the authors, without undue reservation.
